# Enhancing electron diffusion length in narrow-bandgap perovskites for efficient monolithic perovskite tandem solar cells

**DOI:** 10.1038/s41467-019-12513-x

**Published:** 2019-10-03

**Authors:** Zhibin Yang, Zhenhua Yu, Haotong Wei, Xun Xiao, Zhenyi Ni, Bo Chen, Yehao Deng, Severin N. Habisreutinger, Xihan Chen, Kang Wang, Jingjing Zhao, Peter N. Rudd, Joseph J. Berry, Matthew C. Beard, Jinsong Huang

**Affiliations:** 1 Department of Applied Physical Sciences, University of North Carolina, Chapel Hill, NC 27599 USA; 2 Chemistry & Nanoscience Center, National Renewable Energy Lab, Golden, CO 80401 USA

**Keywords:** Devices for energy harvesting, Solar cells

## Abstract

Developing multijunction perovskite solar cells (PSCs) is an attractive route to boost PSC efficiencies to above the single-junction Shockley-Queisser limit. However, commonly used tin-based narrow-bandgap perovskites have shorter carrier diffusion lengths and lower absorption coefficient than lead-based perovskites, limiting the efficiency of perovskite-perovskite tandem solar cells. In this work, we discover that the charge collection efficiency in tin-based PSCs is limited by a short diffusion length of electrons. Adding 0.03 molar percent of cadmium ions into tin-perovskite precursors reduce the background free hole concentration and electron trap density, yielding a long electron diffusion length of 2.72 ± 0.15 µm. It increases the optimized thickness of narrow-bandgap perovskite films to 1000 nm, yielding exceptional stabilized efficiencies of 20.2 and 22.7% for single junction narrow-bandgap PSCs and monolithic perovskite-perovskite tandem cells, respectively. This work provides a promising method to enhance the optoelectronic properties of narrow-bandgap perovskites and unleash the potential of perovskite-perovskite tandem solar cells.

## Abstract (web summary)

Tin-based perovskites possess the suitable narrow-bandgap for tandem solar cells but their short carrier diffusion lengths limit device efficiency. Here Yang *et al*. add cadmium ions to increase diffusion length to above 2 µm by reducing the background free hole concentration and electron trap density.

## Introduction

The power conversion efficiencies (PCEs) of single junction perovskite solar cells (PSCs) have recently rapidly increased to 23.7%^1–7^, approaching the Shockley–Queisser limit^8^. To further enhance PCE of PSCs, an attractive route is to construct multijunction solar cells with multiple absorbing layers, which have complementary absorption spectra. Owing to the tunable bandgap and small open-circuit voltage (*V*_OC_) deficit of perovskite materials^9–11^, they have been successfully coupled with other narrower bandgap semiconductors such as Si^12–14^, CIGS^15,16^, semiconducting polymers^17^ and CZTSSe^18^ to develop tandem solar cells. To date, the PCE of Si-perovskite tandem solar cells has reached certified value of 28.0%, higher than the efficiencies of either single-junction perovskite or Si solar cells^1^. The wide bandgap tunability from 1.20 to 2.30 eV allows the development of perovskite–perovskite tandem solar cells, which still enjoys the benefits of low-cost solution processing of perovskite materials. To date, both four-terminal and monolithic perovskite–perovskite tandem solar cells have been successfully developed. Im and coworkers reported the first monolithic perovskite–perovskite tandem cell with PCE of 10.4%, and the PCE has been gradually increased to 21.0%^19–23^. Jen and coworkers reported the first four terminal perovskite–perovskite tandem cells with an efficiency of 19.1%^24^, and the efficiency has now gone up to 23.1%^25^.

From the perspective of cost, developing monolithic tandem cells is potentially more valuable. However, one main limitation for relatively low PCE of perovskite monolithic tandem solar cells comes from the poor semiconducting properties of Sn-based narrow-bandgap (NBG) perovskites. Till now, Sn substitution in Pb-perovskites is the only approach to reduce the bandgap to as narrow as 1.20 eV. However, compared to Pb-perovskites, Sn-containing NBG perovskites are reported to have higher trap density of states (*t*DOS)^26^, shorter carrier recombination lifetime^27^, larger Urbach energy^28^, higher background carrier concentration due to oxidization of Sn^2+^ ions^29^, resulting in a high *V*_OC_ deficit, and lower absorption coefficient in near infrared region^28^ and low short-circuit current density (*J*_SC_). The smaller absorption coefficient in near infrared region than in visible range in Sn containing NBG perovskites makes the problem even worse, because a much thicker perovskite film is needed to fully absorb the infrared part of sunlight.

To address these issues, many efforts have been devoted to improving the quality of Sn-containing NBG perovskites. Mhaisalkar and Mathews et al. effectively reduced the Sn vacancy density, which might cause the strong self-doping, by adding additive SnF_2_^30^. Jen and coworkers passivated the defeats of Sn containing NBG perovskites by ICBA and got an extremely low *V*_OC_ deficit of 0.33 V^31^. Most recent work by Yan et al. demonstrated enlarging grains and reducing electronic disorder of NBG perovskites using chlorine-containing additives in precursor solution^30^. Nevertheless, the *J*_SC_ of NBG perovskite devices reported so far is still much lower than the theoretical limit. For example, a NBG perovskite with a bandgap of 1.22 eV, which is generally used in perovskite monolithic tandem cells, has a theoretical *J*_SC_ upper limit of 35.1 mA cm^−2^ by assuming an external quantum efficiency (EQE) of 90% on average, while the highest reported *J*_SC_ is less than 30 mA cm^−2^, and increased *J*_SC_ often causes reduction of *V*_OC_ or fill factor (FF)^23,32^.

In this work, we find that the limiting factor for the far-from ideal *J*_SC_ in Sn-containing NBG PSCs is the much shorter electron diffusion length in comparison to the hole diffusion length. We report increasing the electron diffusion length to 2.72 ± 0.15 µm by adding a very small amount of Cd^2+^ ions into the precursor solution, allowing much thicker Sn-containing NBG active layer in PSCs to improve harvesting more infrared emission from sunlight. Employing Cd^2+^ ions enhances the PCE of both NBG single junction solar cells and monolithic all-perovskite tandem solar cells.

## Results

### Simulation of the NBG and monolithic perovskite tandem solar cells

**Fig. 1:**
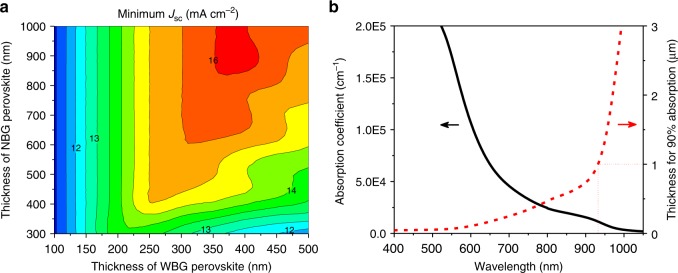
Simulation of the NBG and monolithic perovskite tandem solar cells. **a** Simulated *J*_SC_ of perovskite–perovskite tandem solar cells with assumption of 90% EQE in the whole absorption spectrum. In the simulation, the NBG FA_0.5_MA_0.45_Cs_0.05_Pb_0.5_Sn_0.5_I_3_ and WBG FA_0.6_Cs_0.4_Pb(I_0.65_Br_0.35_)_3_ perovskites have bandgaps of 1.22 eV and 1.80 eV, respectively. **b** Absorption coefficient of FA_0.5_MA_0.45_Cs_0.05_Pb_0.5_Sn_0.5_I_3_ with a bandgap of 1.22 eV, and corresponded thickness of the films that is needed to absorb 90% of different incident light. Source data are provided as a Source Data file

To date, the reported narrowest bandgap of NBG perovskites is ~1.22 eV that gives matched *J*_SC_ with a wide bandgap (WBG) layer of around 1.80 eV bandgap in a monolithic tandem solar cell^21^. Considering the main limitation for the PCE of NBG PSCs is from *J*_SC_, an optical simulation was performed to find out what is the optimal thickness of NBG perovskite layer in the tandem solar cells. Here, FA_0.5_MA_0.45_Cs_0.05_Pb_0.5_Sn_0.5_I_3_ with a bandgap of 1.22 eV and FA_0.6_Cs_0.4_Pb(I_0.65_Br_0.35_)_3_ with a bandgap of 1.80 eV are used for optical simulation in the perovskite–perovskite monolithic tandem solar cells. In the simulation, the *J*_SC_ of tandem cell is determined by the smaller one of the two sub-cells, where the EQEs in the whole absorption spectrum are set to be 90%. As shown in Fig. 1a, the maximum *J*_SC_ achievable for the tandem cell with these two chosen subcell bandgaps is 16.0 mA cm^−2^. In order to reach this maximum *J*_SC_, the NBG perovskite layer needs to be 1000 nm thick so that its photocurrent can match that from the 400 nm thick WBG perovskite layer. Figure 1b shows the absorption coefficient of Cs_0.05_MA_0.45_FA_0.5_Pb_0.5_Sn_0.5_I_3_, which also tells that a minimal thickness of 1000 nm is required for the NBG perovskite to absorb 90% of incident light in the near-infrared region.

### Influence of Cd^2+^ on the performance of the NBG PSCs

**Fig. 2:**
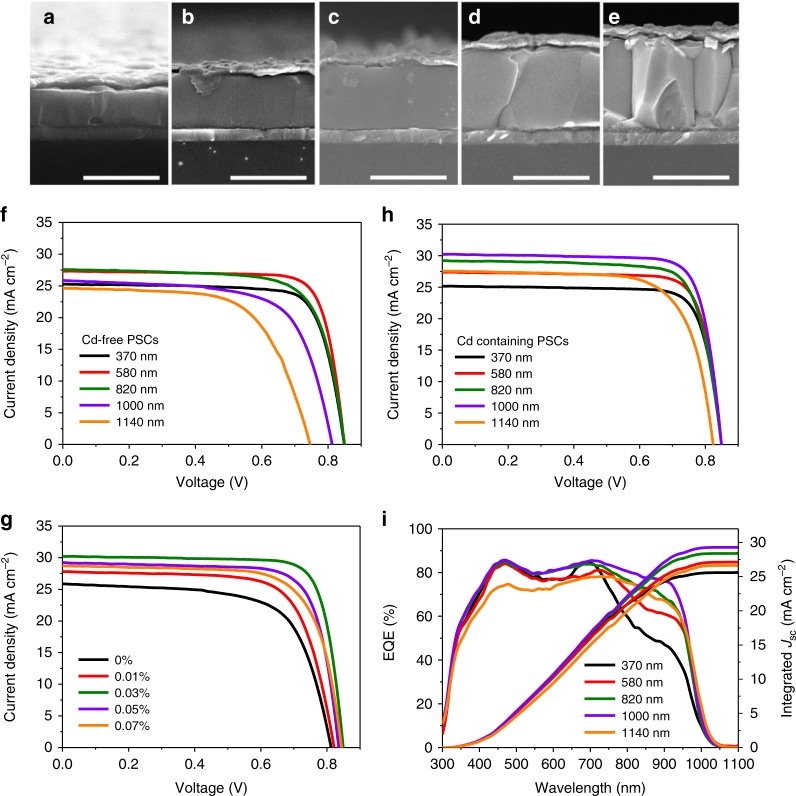
Influence of Cd^2+^ on the performance of the NBG perovskite solar cells. **a**–**e** Cross-sectional SEM images of NBG PSCs without Cd^2+^ ions in different thicknesses of 370 nm (**a**), 580 nm (**b**), 820 nm (**c**), 1000 nm (**d**) and 1140 nm (**e**), respectively. The scale bar is 1 µm in all images. **f** *J–V* characteristics of Cd-free NBG PSCs with different perovskite film thicknesses. **g** *J–V* characteristics of NBG PSCs with vary molar ratio of Cd^2+^ ions. **h** and **i** *J–V* characteristics (**h**) and EQE spectra (**i**) of NBG PSCs that was added with 0.03 mol% Cd^2+^ ions in different perovskite thicknesses. Source data are provided as a Source Data file

Previous studies show that the optimized thickness for NBG cells was 620 nm^32^. Considering perovskites made in different labs might have a variation of film morphology and optoelectronic properties, we first systematically studied the thickness-dependent performance of NBG PSCs with the thicknesses ranging from 370 to 1140 nm as shown in Fig. 2a–e. Figure 2f shows the photocurrent curves of the NBG devices based on these films, and the performances are summarized in Supplementary Table 1. When the thickness of NBG perovskites is increased from 370 to 580 nm, the *J*_SC_ increases from 25.4 to 27.3 mA cm^−2^, resulting an improved PCE from 16.8% to 18.1%. However, as the perovskite thickness is further increased to 1140 nm, the PCEs gradually reduce due to the decrease of *V*_OC_ from 0.85 to 0.75 V, reduced *J*_SC_ from 27.33 to 24.54 mA cm^−2^, and reduced FF from 0.78 to 0.62. This agrees with previously reported results^31^, showing those results are well reproduced.

Surprisingly, we discover that adding trace amount (<0.1 mol%) of Cd^2+^ ions into the precursor solution can significantly enhance the performance of NBG PSCs with 1000 nm thick perovskite layers. To find the optimal concentration of Cd^2+^ ions incorporation, we fixed the NBG film thickness to be 1000 nm, and changed Cd^2+^ ions molar concentration from 0.01 to 0.07%, by tuning the CdI_2_ ratio with PbI_2_ ratio in the precursor films. The *J–V* and EQE curves of the related solar cell devices are shown in Fig. 2g and Supplementary Fig. 1 and the related device performance is summarized in Supplementary Table 2. The optimized concentration of Cd^2+^ ions in the NBG perovskite precursors is found to be 0.03 mol%. We then did a same study of thickness-dependent performance of NBG PSCs with perovskite thicknesses ranging from 370 to 1140 nm and with a fixed Cd^2+^ ion concentration of 0.03 mol% compared to Pb^2+^. The *J–V* curves of these devices are shown in Fig. 2h and device performance is summarized in Supplementary Table 3. When the thicknesses of the NBG perovskite are below 600 nm, the PCEs of the NBG PSC are comparable to those devices without Cd^2+^ ions. Most notably, the *J*_SC_ of the Cd-containing devices continues to increase from 27.35 to 30.24 mA cm^−2^, and the device *V*_OC_ and FF remain unchanged, when the thickness of NBG increases from 580 to 1000 nm. This indicates that the charge collection length is increased in the NBG perovskite by the addition of Cd^2+^ ions. The device with 1000-nm-thick NBG perovskite layer exhibits a record PCE of 20.3% with negligible photocurrent hysteresis (Supplementary Fig. 2) among all reported highly efficient NBG PSCs with a high *V*_OC_ of 0.85 V, *J*_SC_ of 30.24 mA cm^−2^ and FF of 0.79 (The comparison of device performance is shown in Supplementary Table 4). The efficiency is confirmed by the maximum power point output tracking, giving a stabilized PCE of 20.2% (Supplementary Fig. 3). The PCE of NBG PSCs drops slightly when the perovskite thickness is further increased to 1140 nm, which is likely due to the worse morphology of the NBG perovskite films. Figure 2i shows the EQE spectra of the Cd-containing PSCs with perovskite layers of various thickness. The EQE in the near-infrared wavelength region (800–1000 nm) increases when the perovskite thickness is increased from 370 nm to 1000 nm, in agreement with the improved *J*_SC_ from *J*–*V* curves (Fig. 2h). The difference between integrated current density from EQE and *J*_SC_ from *J*–*V* curve is within 3%.

### Electronic properties of NBG perovskites with and without Cd^2+^ ions

**Fig. 3:**
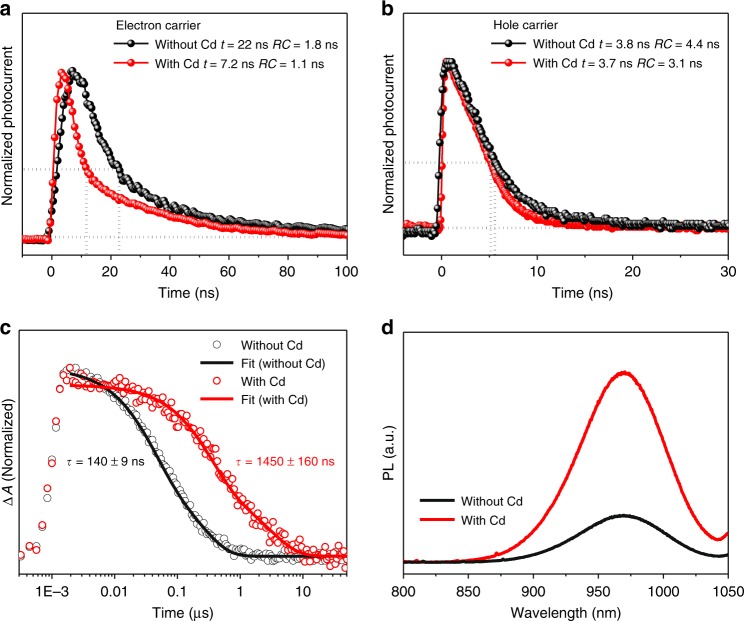
Influence of Cd^2+^ ions on the carrier properties of NBG perovskites. **a** and **b** Normalized transient photocurrent of NBG PSCs with and without Cd^2+^ ions for electron (**a**) and hole (**b**) carriers. **c** TA kinetics near the exciton bleach (950 nm) of NBG perovskites with and without Cd^2+^ ions. *τ* is the average lifetime described in the method. **d** Steady-state PL of 1000-nm-thick NBG perovskite films with and without Cd^2+^ ions. All studies in Fig. 3 are based on 1000-nm-thick perovskite film. Source data are provided as a Source Data file

To gain insight to the mechanism for improved performance in Cd-containing NBG PSCs, carrier diffusion length of electrons and holes in the NBG perovskites with and without Cd^2+^ ions were compared by measuring the carrier mobility and recombination lifetime. The mobilities of electrons and holes in the NBG perovskites with and without Cd^2+^ ions were measured via transient photocurrent decay (TPC), which characterize the charge transport process in real operational devices. The device structures and measurement setup are illustrated in Supplementary Fig. 4. The thicknesses of charge transport layers are so small that they do not impact the measured transit time^33^ (see part of transient photovoltage decay (TPV) and TPC measurement in the methods). It should be noted that the measurement of carrier mobility by TPC method needs to ensure the resistance–capacitance (RC) constant of the circuitry to be small enough so that it does not limit the charge transit time determination. Here we control the RC constant by decreasing device capacitance as summarized in Supplementary Tables 5 and 6, which is realized via reducing the device active area with a laser scriber. Taking the electron mobility measurement as a sample, we gradually reduce the RC value of a Cd-free NBG PSC by reducing the device area and measure the transit time. As shown by the TPC curves in Supplementary Fig. 5, when the RC value is reduced from 101.0 to 49.0 ns, a reduction of transit time was clearly observed, despite the measurement being conducted using a same device. It indicates the transit time is limited by the RC constant in the large area devices. So, we further gradually reduced the RC value to 1.8 ns, while we found the measured transit time reduced and stabilized at 22.0 ns, which is determined to be the real transit time. Figure 3a shows that addition of Cd^2+^ ions reduces the electron transit time by around threefold from 22.0 ns to 7.2 ns in the devices with same thickness of NBG perovskites, which corresponds to an increase of electron mobility from 0.65 cm^2^ V^−1^ s^−1^ to 1.98 cm^2^ V^−1^ s^−1^. However, the TPC measurement for hole carriers are still limited by *RC* constant, although it has been reduced to 3.1 ns, as shown in Fig. 3b. We can still estimate that the hole mobilities are at least 3.86 and 3.25 cm^2^ V^−1^ s^−1^ in the NBG perovskites with and without Cd^2+^ ions, respectively.

The carrier recombination lifetime in the perovskite films was measured by transient absorption (TA) spectroscopy. Figure 3c displays the normalized TA kinetics probed at the center of the ground-state bleach (~950 nm) after 500-nm excitation. The average carrier lifetime of the control NBG perovskite film is 140 ± 9 ns, while it is significantly increased to 1450 ± 160 ns after adding Cd^2+^ ions into the perovskite films, comparable to the best recently reported lifetime^34^. This agrees with the enhanced photoluminescence (PL) intensity in the Cd-containing perovskite films, as shown in Fig. 3d. The carrier recombination process in the PSCs was also measured by the TPV method, though the measured lifetime is also affected by charge combination at interfaces in the devices. As shown by the TPV curves in Supplementary Fig. 6, the Cd-containing PSCs also have doubled charge recombination lifetime compared to the control device, indicating the charge recombination inside the perovskite films dominates. All carrier mobility, recombination lifetime and diffusion length are summarized in Table 1. The calculated electron diffusion length of Cd-free PSCs is only 0.49 ± 0.10 μm, which explains the decreased device PCE when the perovskite thickness is increased to beyond 600 nm. The electron diffusion length is significantly improved to 2.72 ± 0.15 μm after adding Cd^2+^ ions into the perovskite layer, more than fivefold of the value from the Cd-free device. The long electron diffusion length enables efficient electron extraction in 1000-nm-thick perovskite film, affording the high performance in thick Cd-containing PSCs. On the other hand, the hole diffusion length in NBG perovskites with and without Cd^2+^ ions are calculated to be more than 3.80 ± 0.20 and 1.08 ± 0.10 μm, respectively, indicating the hole extraction is not a limiting factor in the NBG PSCs with 1000-nm-thick perovskite layer. Although there has been speculation in the past on the short carrier diffusion length in Sn-perovskites, there is no study to identify what the limiting factor is. Here we conclude the short electron diffusion length is the key limiting factor hindering the performance enhancement of NBG PSCs.

**Table 1:** Carrier mobility, recombination lifetime and diffusion length in 1000-nm-thick NBG PSCs with and without 0.03% Cd^2+^ ions

Species	NBG PSCs (*E*g = 1.22 eV)	Mobility (cm^2^ V^−1^ s^−1^)	Recombination lifetime (ns)	Diffusion length (μm)
Electron	Without Cd^2+^	0.65	140 ± 9	0.49 ± 0.10
Electron	With Cd^2+^	1.98	1450 ± 160	2.72 ± 0.15
Hole	Without Cd^2+^	>3.25	140 ± 9	>1.08 ± 0.10
Hole	With Cd^2+^	>3.86	1450 ± 160	>3.80 ± 0.20

To understand the origin of improved electron diffusion length, we first compared the morphology of perovskite films with and without Cd^2+^ ions with a concentration of 0.03 mol% by scanning electron microscopy (SEM). As shown in the cross-sectional and top view SEM images in Supplementary Figs. 7 and 8f–j, the grain sizes of Cd-containing films are similar with those of Cd-free perovskite films (Fig. 2a–e and Supplementary Fig. 8a–e), which is not surprising because of the very small amount of Cd^2+^ ions added. It indicates the enhanced electron diffusion length is not originated from a change in morphology. In addition, no obvious change of lattice constant, optical properties and photostability of the perovskite films have been observed, again consistent with the small amount of Cd^2+^ ions added (Supplementary Figs. 9–11). Despite the small amount, their existence of Cd^2+^ in the NBG perovskite films is confirmed by X-ray photoelectron spectroscopy (XPS) study. As shown in Supplementary Fig. 12, a characteristic peak of Cd^2+^ 3d clearly shows up in the perovskite film with Cd^2+^, in contract to the control Cd-free NBG perovskites.

**Fig. 4:**
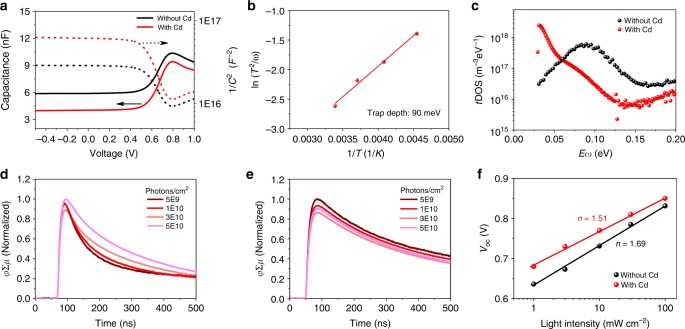
Influence of Cd^2+^ ions on the carrier concentration and *t*DOSs. **a** Capacitance–voltage curves of NBG PSCs with and without Cd^2+^ ions at 295 K. **b** ln(*T*^2^/**ω**) vs 1/*T* curve for determining the trap depth and attempt-to-escape angular frequency. **c** *t*DOS studies of NBG PSCs with and without Cd^2+^ ions at 295 K. **d** and **e** TRMC measurement for electron carrier property with a structure of quartz/PEDOT:PSS/NBG perovskite films without (**d**) and with (**e**) Cd^2+^ ions. The intensities are normalized to the highest peak intensity. **f** *V*_OC_ evolution with light intensity of NBG PSCs with and without Cd^2+^ ions. All studies in Fig. 4 are based on 1000-nm-thick perovskite film. Source data are provided as a Source Data file

The two major factors that determine minority carrier recombination process in a semiconductor are background majority carrier concentration and non-radiative trap density. Therefore, the background majority carrier concentration of the NBG perovskite was measured with a capacitance–voltage method. As shown in Fig. 4a, the measured background carrier concentration of the NBG perovskite decreases from 1.0 × 10^16^ cm^−3^ to 4.2 × 10^15^ cm^−3^ after adding Cd^2+^ ions, which indicates that Cd^2+^ ions de-dope the NBG perovskite. Previous studies revealed Sn vacancies are the most abundant intrinsic defects in Sn-based perovskites^26^, which induces self-doping of Sn perovskite. Cd^2+^ ions with a relative smaller ionic radius are reported to incorporate into the lattice of Pb-based perovskites to reduce internal strain ^35^. Therefore, we propose that the Cd^2+^ ions can fill the Sn vacancies to de-dope Sn perovskite, which enhances minority carrier recombination lifetime, carrier mobility and diffusion length.

Temperature-dependent thermal admittance spectroscopy was conducted to determine the *t*DOS and depth of traps with a regular solar cell structure. A clear trap state with average activation energy of 90 meV can be derived from the temperature-dependent capacitance–frequency measurement in the Cd-free perovskite devices as shown in Fig. 4b and Supplementary Fig. 13. The density of this trap state at 295 K reduces by more than one order of magnitude after adding Cd^2+^ ions as shown in Fig. 4c, indicating an excellent passivation effect of Cd^2+^ ions. The reduction of trap density by Cd^2+^ ions is further confirmed by time-resolved microwave conductance (TRMC) study with a varied incident light intensity. Using a sample structure of quartz/charge-accepting layers/perovskite, the effect of Cd^2+^ ions on change of electron and hole trap density can be determined. Here poly(3,4-ethylenedioxythiophene):poly(styrene-sulfonate) (PEDOT:PSS) and [6,6]-phenyl-C_61_-butyric acid methyl ester (PCBM) are inserted to extract photogenerated holes and electrons, respectively. As shown in Fig. 4d, the electron recombination lifetimes of Cd-free film gradually increase with the increasing incident light intensity, which suggests the existence of electron traps. While the electron recombination lifetime of Cd-containing film almost maintains the same at varied exciting light intensity, as shown in Fig. 4e. For the films with PCBM, the hole recombination kinetics is found to be largely unchanged, as shown in Supplementary Fig. 14. This proves that adding Cd^2+^ ions into the NBG perovskite mainly reduces the density of electron traps. This result agrees well with the *t*DOS measurement and reveals the mechanism for the improved electron carrier mobility. Filling of charge traps will generally affect diode ideality factor because of charged charge recombination mechanism. Therefore, we conducted light-intensity-dependent *J*–V measurements to derive the ideality factor of the PSCs, since the charge recombination behavior can be indicated by the device ideality factor^36^. As seen in Fig. 4f, the drop of ideality factor by adding Cd^2+^ ions is in consistent with the improved optoelectronic properties. We conclude that adding Cd^2+^ ions not only de-dopes the NBG perovskite, but also reduces the electron trap density, which enhances minority carrier mobility, recombination lifetime and diffusion length.

### Photovoltaic performance of tandem solar cells

**Fig. 5:**
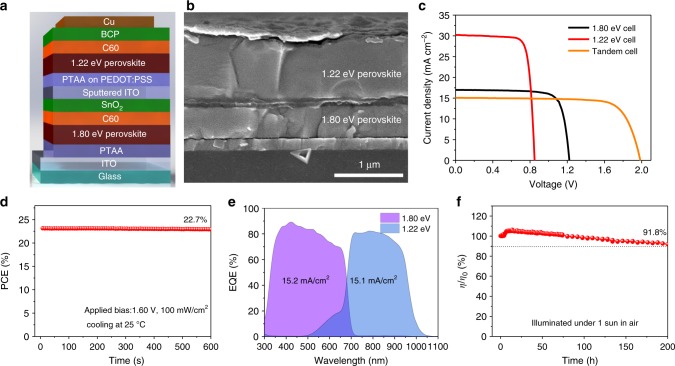
Perovskite–perovskite monolithic tandem cells with Cd^2+^ ions in NBG perovskites. **a**, **b** Illustration and cross-sectional SEM image of a perovskite–perovskite tandem solar cell. **c** *J–V* characteristics of tandem solar cell and individual WBG and NBG single junction PSCs. **d** Steady-state output of tandem solar cells at the maximum power point (applied bias of 1.60 V) under AM1.5 illumination at 25 °C. **e** Individual EQE spectra of WBG and NBG sub-cells in the tandem solar cells. **f** Long-term photo stability test of the tandem solar cells under continuous AM1.5 illumination. *η* and *η*_0_ represent the evaluated and initial PCEs, respectively. Source data are provided as a Source Data file

Finally, this Cd-containing NBG perovskite was used in perovskite–perovskite monolithic tandem solar cells with a structure of ITO/PTAA/FA_0.6_Cs_0.4_Pb(I_0.65_Br_0.35_)_3_ (1.80 eV)/C_60_/SnO_2_/ITO/PEDOT:PSS/PTAA/Cd-FA_0.5_MA_0.45_Cs_0.05_Pb_0.5_Sn_0.5_I_3_/C_60_/BCP/Cu shown in Fig. 5a, b. The 1000-nm-thick NBG perovskite and 400 nm WBG perovskite were applied in the tandem structure to maximize the *J*_SC_ according to our optical simulation results shown in Fig. 1a. The best single junction WBG PSC has a PCE of 16.3% with a *V*_OC_ of 1.22 V, *J*_SC_ of 17.0 mA cm^−2^ and FF of 0.78 (Fig. 5c and Table 2). When combined with the optimized Cd-containing NBG PSC, the best tandem solar cell achieves a highest PCE of 23.0% under reverse scan with a *V*_OC_ of 1.99 V, a *J*_SC_ of 15.1 mA cm^−2^ and FF of 0.77 as shown in Fig. 5c and S15. The PCE of the tandem cells was further confirmed by tracking steady-state output at maximum power point. A stabilized PCE of 22.7% is observed after continuous illumination for 600 s (Fig. 5d). To understand the current matching between WBG and NBG sub-cells in the tandem solar cells, EQE spectra of sub-cells were measured and are shown in Fig. 5e. The integrated *J*_SC_ of WBG and NBG sub-cells are 15.2 and 15.1 mA cm^−2^, respectively, which agree with the *J*_SC_ from *J–V* measurements. The optical simulation further confirms the front WBG subcell does not affect the light absorption of the back NBG subcell in a tandem structure as shown in Supplementary Figs. 16 and 17. Finally, the photostability of encapsulated tandem cells is measured in ambient condition with humidity of 30–50%. As shown in Fig. 5f, a tandem cell maintains 91.8% of their initial PCE after being illuminated under 1 sun for 200 h. PCE statistics for tandem solar cells is shown in Supplementary Fig. 18 and Supplementary Table 7.

**Table 2:** *J–V* characteristics of individual WBG and NBG single-junction PSCs and their tandem solar cells measured under AM1.5 illumination

Cell type	*V*_OC_ (V)	*J*_SC_ (mA cm^−2^)	FF	PCE (%)
Single junction, bandgap of 1.80 eV	1.21 ± 0.03 (1.22)	16.5 ± 0.6 (17.0)	0.77 ± 0.2 (0.78)	15.5 ± 0.5 (16.3)
Single junction, bandgap of 1.22 eV	0.83 ± 0.03 (0.85)	29.7 ± 0.6 (30.2)	0.78 ± 0.02 (0.79)	19.4 ± 0.6 (20.3)
Tandem solar cells	1.95 ± 0.02 (1.99)	15.0 ± 0.3 (15.1)	0.76 ± 0.2 (0.77)	22.2 ± 0.4 (23.0)

Average values with standard deviation. The data for single junction and tandem devices was obtained from 20 and 50 devices, respectively. Parameters of the best cell are reported in brackets

## Discussion

In conclusion, we find that the short electron diffusion length is the main limitation factor in realizing a highly efficient Sn-based NBG PSCs. The electron diffusion length is effectively enhanced by three times to 2.72 ± 0.15 µm by adding 0.03 mol% of Cd^2+^ ions in the perovskite, which enables much thicker Sn-perovskite layers for harvesting more infrared light. The NBG PSCs with optimal perovskite thickness of 1000 nm demonstrate exceptional stabilized efficiencies of 20.2%. By coupling with a WBG perovskite, a high PCE of 22.7% is achieved in a monolithic perovskite–perovskite tandem cell. This work provides a promising method to enhance the optoelectronic properties of NBG perovskites, which unlock the potential of high-efficiency, low-cost and solution-processable perovskite–perovskite tandem solar cells.

## Methods

### Materials

Formamidine iodide, methylammonium iodide were purchased from Greatcell Solar company. PbI_2_ (99.999%), PbBr_2_ (99.999%), SnI_2_ (99.999%), CdI_2_ (99.999%), SnF_2_ (99%), CsI (99.999%), *N*,*N*-dimethylformamide (99.8%, anhydrous), dimethyl sulfoxide (99.8%, anhydrous), 1,2-dichlorobenzene (99%), toluene (anhydrous, 99.8%), poly(bis(4-phenyl)(2,4,6-trimethylphenyl)amine)(PTAA) and bathocuproine (BCP) were purchased from Sigma-Aldrich. Acetone and isopropanol alcohol were purchased from VWR company. PCBM and C_60_ were purchased from NANO-C company. PEDOT:PSS (Clevios™ P VP AI 4083) was purchased from Heraues company.

### Preparation of perovskite precursor solutions

A total of 1.35 M WBG perovskite FA_0.6_Cs_0.4_Pb(I_0.65_Br_0.35_)_3_ precursor solution was prepared by dissolving 0.139 g of CH(NH_2_)_2_I, 0.140 g of CsI, 0.249 g of PbI_2_ and 0.297 g of PbBr_2_ in a mixed solvent of DMSO and DMF with a volume ratio of 3:7. NBG perovskite FA_0.5_MA_0.45_Cs_0.05_Pb_0.5_Sn_0.5_I_3_ precursor solution with a concentration of 1.2–2.1 M was prepared by dissolving 0.45 mol of MAI, 0.5 mol of FAI, 0.05 mol of CsI, 0.55 mol of PbI_2_, 0.5 mol of SnI_2_ and 0.05 mol of SnF_2_ in a mixed solvent of DMSO and DMF with a volume ratio of 3:7. A total of 0.2 M CdI_2_ in DMF is prepared as additive for perovskite precursors. All the perovskite solutions were filtered with polytetrafluoroethylene (PTFE) filters (0.22 μm) before use. The NBG perovskite films with thicknesses of 370, 580, 820, 1000 and 1140 nm are prepared by spin-coating 1.3 M precursor solution under 5 K r.p.m., 1.6 M precursor solution under 5 K r.p.m., 1.6 M precursor solution under 3.5 K r.p.m., 1.8 M precursor solution under 3.5 K r.p.m. and 2.0 M precursor solution under 3.5 K r.p.m., respectively.

### Device fabrication

Patterned ITO glass substrates were cleaned by sequential ultrasonication in acetone and isopropyl alcohol for 30 min. Then, the ITO substrates were further cleaned via UV ozone treatment for 20 min. For the fabrication of NBG PSCs, PEDOT:PSS solution was spin-coated onto the ITO substrates at 5000 r.p.m. for 30 s and anneal at 150 °C for 10 min in ambient condition. After the substrates were transfer into the glovebox, a diluted 0.5 mg mL^−1^ PTAA solution was spin-coated at a speed of 8000 r.p.m. for 30 s and annealed at 100 °C for 10 min. Then 50 μL of NBG perovskite precursor solution was spin-coated for 30 s; 0.3 mL of PCBM solution in toluene (1 mg mL^−1^) was quickly dropped onto the spinning substrate after 10 s elapsed, followed by annealing at 100 °C for 10 min. The thicknesses were controlled by tuning the concentration of precursor solution and spin coating speed. Finally, 20 nm C_60_, 6 nm BCP and 80 nm Cu were sequentially thermal evaporated onto the perovskite films to complete the fabrication of single junction NBG PSCs. For the fabrication of tandem solar cells, 2 mg mL^−1^ PTAA solution was spin-coated onto the ITO substrates at the speed of 5000 r.p.m. for 30 s, followed by annealing at 100 °C for 10 min. After the PTAA-coated substrates were pre-wetted by spinning 50 μL of DMF at 5000 r.p.m. for 10 s, 50 μL of WBG perovskite precursor solution was spin-coated onto PTAA at 5000 r.p.m. for 20 s and 1000 r.p.m. for 20 s. A nitrogen flow is blown onto the substrate for fast drying in the 20th second. Next, the samples were annealed at 65 °C for 10 min and 100 °C for 10 min. After thermal evaporating 30 nm C_60_ onto the perovskite films, 13 nm SnO_2_ film was deposited by atomic layer deposition method under vacuum of 0.2 mTorr at 100 °C with [(CH_3_)_2_N]_4_Sn and H_2_O as Sn and O sources. Afterward, 10 nm ITO was sputtered onto SnO_2_ as recombination layer by a Lesker Sputter system (PRO Line™ PVD 75™) with low power of 100 W (RF mode) under a 3 mTorr mixed gas of Ar and O_2_ at room temperature. Soon after, PEDOT:PSS solution was spin-coated onto the ITO substrates at 5000 r.p.m. for 30 s and anneal at 100 °C for 20 min in ambient condition. Then repeating the fabrication procedure of NBG PSCs as described above to complete the tandem solar cells. Different solar cells in one device substrate were separated by laser scribing (Resonetics Rapid X250 laser ablation system) for avoiding the connection in the middle-sputtered ITO layer. The device areas of all solar cells are 8 mm^2^.

### Device and material characterizations

*J–V measurement*: Simulated AM 1.5 G irradiation was produced by a Newport Oriel Sol3A solar simulator (Oriel 94943A, 450 W) with an AM1.5 filter. The light intensity was calibrated by a reference Si solar cells and meter (P/N 5110V, Newport). *J–V* measurements of solar cells are performed by a Keithley 2400 Source meter under AM 1.5 G illumination at reverse scan mode with a scan rate of 0.05 V s^−1^. For the long-term stability measurement, the tandem devices were encapsulated with Gorilla clear epoxy and slide glass and connected to a resistor so that it can operate at its maximum power point under illumination. Then it was characterized under constant AM 1.5G illumination in ambient condition with a relative humidity between 30% and 50% at room temperature that was maintained by a cooling stage. All single junction and tandem devices were measured by applying a shadow mask with an aperture area of 6.84 mm^2^.

*EQE measurements*: EQE measurements were conducted with a Newport QE measurement kit by focusing a monochromatic beam of a Bruker Vertex 80v Fourier Transform Interferometer with tungsten lamp source onto the devices. Then the photocurrent was obtained through Stanford Research SR570 current preamplifier. EQE of devices were calibrated by a Newport reference silicon solar cell with a known EQE. The EQE of WBG and NBG sub-cells in tandem cells were measured by respectively exposing the tandem cell under a 470-nm and 940-nm LED lamp for saturating the other junction during measurement.

*TPV and TPC measurements*: For the TPV measurements, the solar cells were connected to a digital oscilloscope (DOS-X 3104A) and illuminated by a solar simulator with 1 sun intensity to form an open-circuit condition (the internal impedance of the oscilloscope was set to 1 MΩ). An attenuated 337 nm laser pulse (SRS NL 100 Nitrogen Laser, frequency of 10 Hz and pulse width of <3.5 ns.) was applied as a small perturbation to the 1 sun background illumination on the device. The laser-pulse induces a photovoltage variation (Δ*V*) to the *V*_OC_ that produced by 1 sun background illumination. For the TPC measurement, the device was also connected to the oscilloscope and the internal impedance was set to 50 Ω to form a short-circuit condition. A photocurrent variation (Δ*I*) is produced by a same laser pulse that is used in TPV measurement. No background illumination is used in TPC measurement. The carrier mobility *μ* were calculated from the relationship of *μ* = *d*^2^/*tV*_bi_, where *d*, *V*_bi_ and *t* are transit distance, built-in potential and transit time, respectively^6^. We calculated charge transit time to verify which layer is the limiting *J*_SC_ transit time. The thickness and mobility of C_60_ is just 20 nm, and the mobility is about 0.1– to 1 cm^2^ V^−1^ s^−1^. The diffuse time of carrier transport through C_60_ layer is calculated to be 0.15–1.5 ns. The real transit time is even shorter, because of the additional built-in electric field. This transit time is far smaller than what we measured in the TPC measurement. Therefore, the electron transit time is not limited by the C_60_ layer.

*tDOS measurement:* Temperature-dependent thermal admittance spectroscopy is used to measure the energetic profile of trap density, and the *t*DOS of the devices (*N*_T_) are calculated by the equation $N_T\left( {E_{\mathbf{\omega}} } \right) = - \frac{1}{{qk_{\mathrm{B}}T}}\frac{{{\mathbf{\omega }}dC}}{{d{\mathbf{\omega }}}}\frac{{V_{{\mathrm{bi}}}}}{W}$, where *W* and *V*_bi_ are the depletion width and build-in potential, respectively, that were derived from Mott–Schottky analysis; *q*, *k*_B_, *T*, **ω** and *C* are elementary charge, Boltzmann’s constant, temperature, angular frequency and capacitance, respectively^37^. An energetic demarcation is defined by the applied angular frequency ω: $E_\omega = k_{\mathrm{B}}T{\mathrm{ln}}\frac{{{\boldsymbol{\omega }}_0}}{{\mathbf{\omega }}}$, where *ω*_0_ is the attempt-to-escape angular frequency. The activation energy of trap states and attempt-to-escape frequency are calculated to be 90 meV and 3.76 MHz according to the relationship ${\mathrm{ln}}\left( {\frac{{T^2}}{{\mathbf{\omega }}}} \right) = \frac{{E_{\mathrm{T}}}}{{kT}} - {\mathrm{ln}}(2\pi v_0)$, where *T*, **ω**, *E*_T_, *k*, *ν*_0_ are temperature, angular frequency, activation energy of trap states, Boltzmann constant and attempt-to-escape frequency, respectively. For the depletion width of devices in vary temperatures, according to the capacitance–voltage measurement at 295 K as shown in Fig. 3i, the capacitance does not change when there is a reverse bias, indicating the device is fully depleted, i.e. the depletion width is equal to the thickness of perovskite film, which is ~1 μm. The depletion width increases when the temperature decreases according to their relationship $W_D = \sqrt {\frac{{2\varepsilon }}{{qN}}(V_{{\mathrm{bi}}} - V - \frac{{2kT}}{q})}$, mainly due to reduced dark carrier density. Therefore, the depletion widths measured at all temperatures from 220 to 295 K in this work are about 1 μm. The trap states below the energy demarcation are able to capture or emit carriers with the given *ω* and contribute to the capacitance.

*TA measurement*: Microsecond TA spectra were collected using a Helios spectrometer (Ultrafast systems). A Coherent Libra regeneratively amplified Ti:sapphire laser with ∼4 W, 1 kHz and ∼150 fs pulse-width output at 800 nm was used for pump-beam generation. The 800-nm beam was directed into a TOPAS optical parametric amplifier to generate a pump pulse at 500 nm. The probe beam is derived from an EOS system and was electronically delayed with respect to pump laser pulse. The probe beam produced was a broadband near infrared spectrum from 850 to 1600 nm. The probe was then passed through a continuously variable neutral-density filter and a fraction was separated off to be used as a reference that accounts for probe-beam intensity fluctuations. The pump and probe beams were then overlapped at the sample. Near infrared photodiode arrays (Ultrafast Systems) were used to detect the probe and reference beams for data acquisition. To extract the lifetime of charge carriers, the transient kinetics is modeled with a bi-exponential decay function and the average lifetime (*τ*) is reported here as $\tau = \frac{{A_1\tau _1 \, + \, A_2\tau _2}}{{A_1 \, + \, A_2}}$.

*TRMC*: For the TRMC measurements, the perovskite films were deposited onto quartz substrates (2.75 cm^2^ area). The samples are pumped with a 5-ns pulse width beam (640 nm) from an OPO pumped by the third harmonic of an Nd:YAG laser, and probed by microwaves at around 9 GHz. The microwave field is absorbed by photogenerated mobile carriers in the NCs, and its relative change in power ∆*P* can be measured. The change in microwave power relates to the photoconductivity ∆*G* through Δ*P*/*P* = −*K*Δ*G*, where *K* is an empirically determined calibration factor for the microwave cavity used in this experiment. The photoconductivity is proportional to the number of charges and their mobility. It can be expressed as Δ*G* = *eβF*_A_*I*_0_(*ϕ*∑*μ*), where *e* is the elementary charge, *β* = 2.2 is the geometric factor for the X-band waveguide used, *I*_0_ is the incident photon flux, *F*_A_ the fraction of light absorbed at the excitation wavelength, *ϕ* is the quantum efficiency of free carrier generation per photon absorbed and ∑*μ* = *µ*_e_ *+* *µ*_h_ the sum of the mobilities of electrons and holes. Bi-exponential fits of the photoconductivity decay transients were weighted to calculate the average carrier lifetime using the equation: *τ*_avg_ = (*A*_0_*τ*_0_ + *A*_1_*τ*_1_)/(*A*_0_ + *A*_1_). For a charge-carrier yield of *f* *=* 1, the combined charge carrier mobility at *t* = 0 can be derived from the sum of the pre-exponential factors (∑*A*) of the fits.

*Capacitance–voltage measurement*: Capacitance–voltage measurement was conducted with an Agilent E4980A LCR meter. The carrier concentration *n* can be derived from the equation $n = \frac{2}{{q\varepsilon A^2d(1/C^2)/dV}}$, where *n*, *q*, *ε*, *A*, *C* and *V* are carrier concentration, element charge, dielectric constant, device area, capacitance and applied voltage, respectively.

*Optical simulations*: The thickness at 90% absorption in Fig. 1a is derived from another format of Beer–Lambert law *ℓ*=−ln10 × (lg*T*)/*a*, where *T* and *a* is the transmittance and absorption coefficient. A Matlab program based on transfer matrix method was used for optical simulations in Fig. 1b^38^. Theoretical photocurrent in optical simulation is obtained assuming 90% EQE for both WBG and NBG perovskites in absorbing region.

*Other characterizations*: The SEM images were taken from a Hitachi S-4700 Cold Cathode Field Emission Scanning Electron Microscope. Light-intensity-dependent measurements for ideality factors were measured with a series of neutral optical density filters. Film thicknesses were measured by a Bruker DektakXT stylus profiler. PL spectra were measured with a Horiba iHR320 Imaging Spectrometer. X-ray diffraction (XRD) patterns were measured with a Rigaku Miniflex 6GBenchtop XRD system. UV-vis absorbance is measured with Evolution™ 201 UV-Visible Spectrophotometers. XPS is conducted by Kratos Axis Ultra DLD X-ray Photoelectron Spectrometer with a monochromatic Al K alpha source for high-energy resolution work.

### Reporting summary

Further information on research design is available in the Nature Research Reporting Summary linked to this article.

## Supplementary information

Supplementary Information

Solar Cells Reporting Summary

Source Data

## Data Availability

The data that support the findings of this study are available from the corresponding author upon reasonable request.
