# Supplementary material for: Enhancing electron diffusion length in narrow-bandgap perovskites for efficient monolithic perovskite tandem solar cells
Source: Nat Commun. 2019 Oct 3;10:4498. doi: 10.1038/s41467-019-12513-x (PMC6776504; doi:10.1038/s41467-019-12513-x)
Supplement: Supplementary file 1 — Supplementary Information [file 41467_2019_12513_MOESM1_ESM.pdf]

Supplementary information for

Enhancing Electron Diffusion Length in Narrow-bandgap Perovskites for Efficient Monolithic Perovskite Tandem Solar Cells

Yang et al.

Supplementary Figure 1-18

Supplementary Table 1-7

Supplementary References

Third part test report

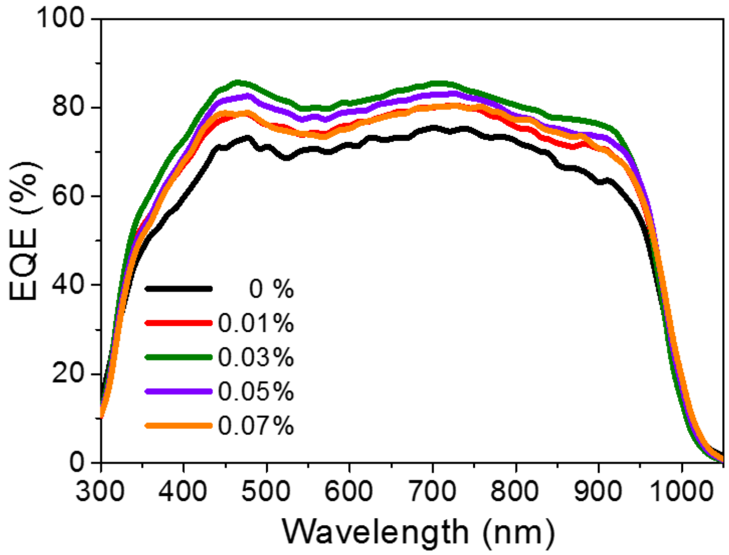

Supplementary Figure 1. EQE spectra of NBG PSCs with vary molar ratio of Cd^{2+} ions.

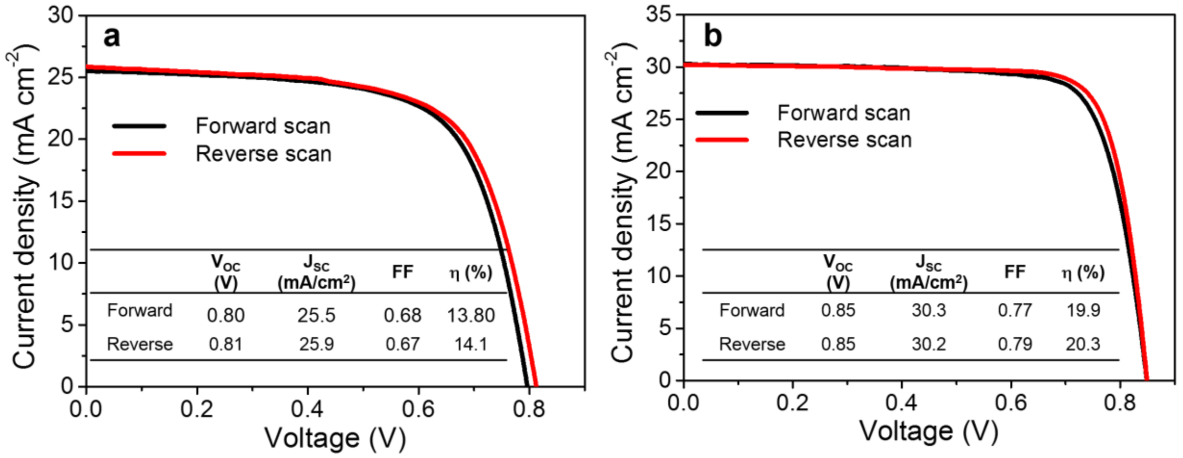

Supplementary Figure 2. Hysteresis study of the NBG PSCs without (**a**) and with (**b**) Cd^{2+} ions. The thickness of perovskite layer in the solar cells are 1000 nm.

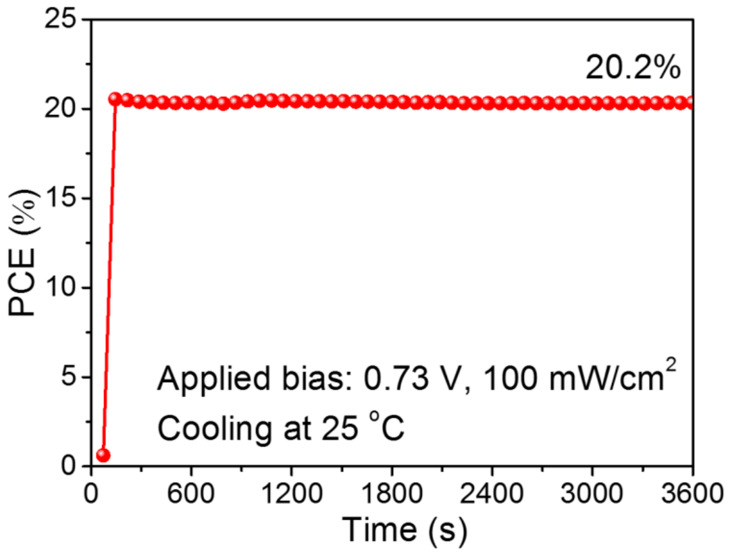

Supplementary Figure 3. Steady-state output of NBG PSCs with 0.03% Cd^{2+} ions, which were measured at the maximum power point (applied bias of 0.73 V) under constant AM1.5 illumination at 25 °C.

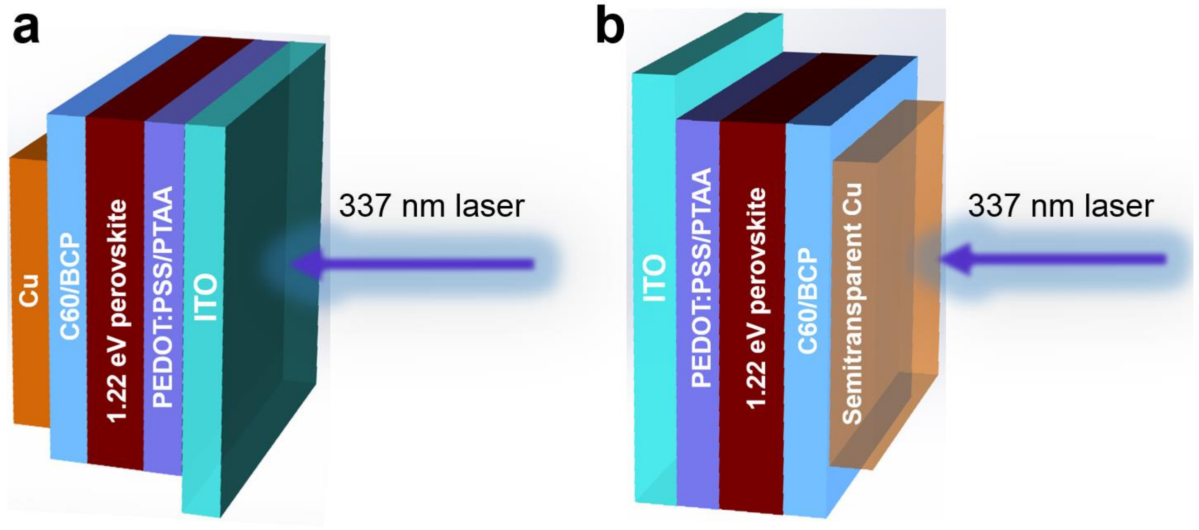

Supplementary Figure 4. Laser incident paths of TPC studies for electron (**a**) and hole (**b**) carrier behaviors, respectively.

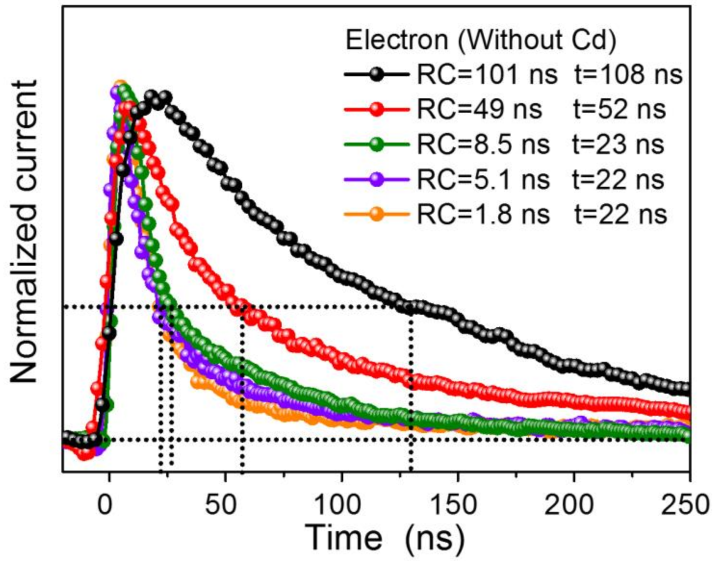

Supplementary Figure 5. Systematical TPC study of a Cd-free NBG PSC with different device areas in the same device. The device area is gradually reduced by a laser scriber. The perovskite film thickness in the NBG PSC is 1000 nm.

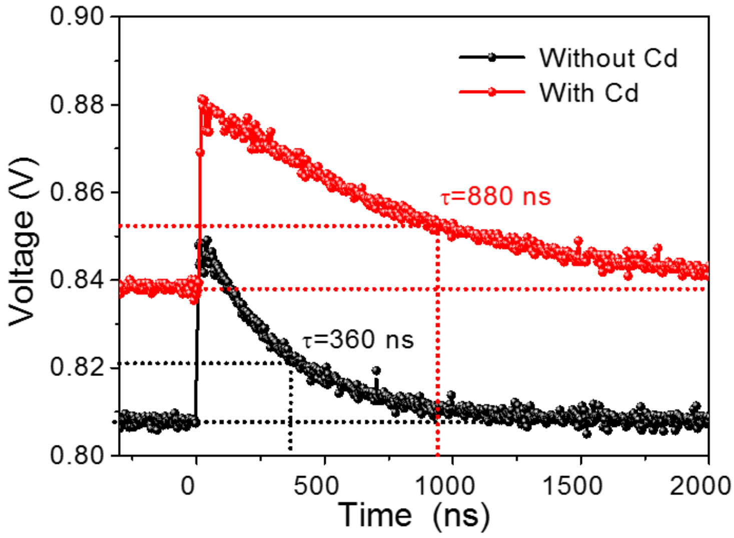

Supplementary Figure 6. TPV study of NBG PSCs with and without Cd^{2+} ions.

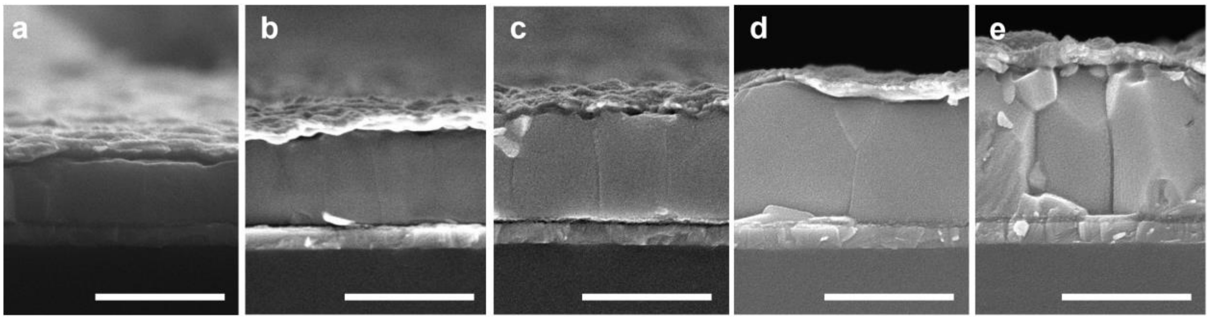

Supplementary Figure 7. Cross-sectional SEM images of NBG PSCs with 0.03% Cd^{2+} ions in different thicknesses of 370 nm (a), 580 nm (b), 820 nm (c), 1000 nm (d) and 1140 nm (e), respectively. The scale bar is 1 μm in all images.

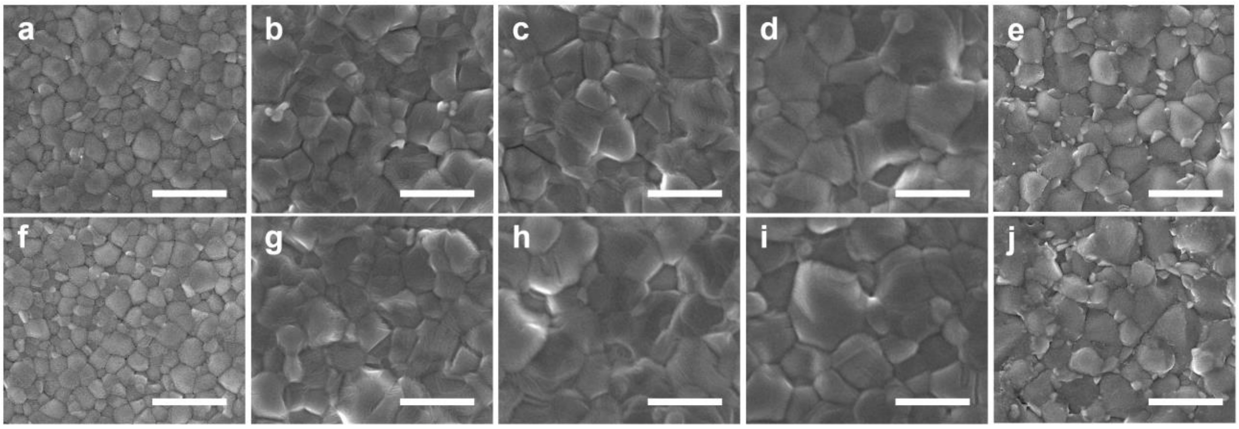

Supplementary Figure 8. Top-view SEM images of NBG PSCs without (a-e) and with (f-j) Cd^{2+} ions in different thicknesses of 370 nm (a, f), 580 nm (b, g), 820 nm (c, h), 1000 nm (d, i) and 1140 nm (e, j), respectively. The scale bar is 1 µm in all images.

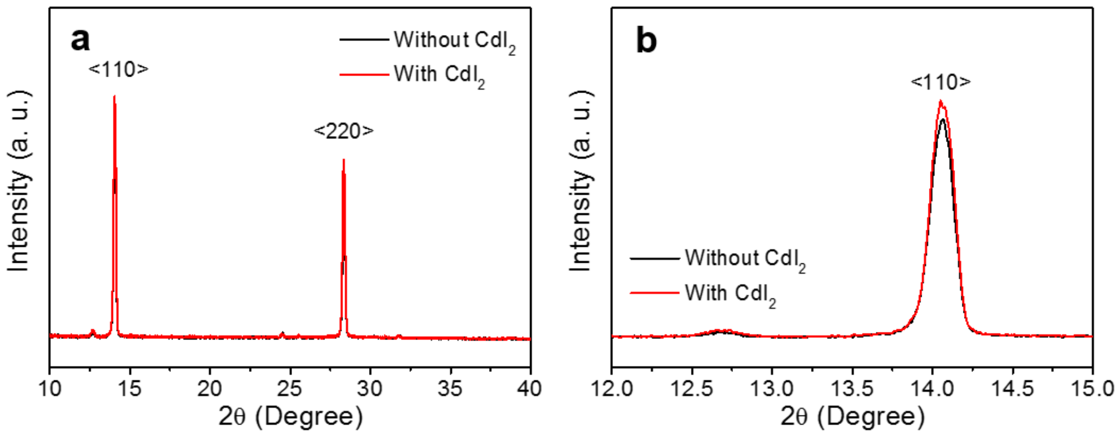

Supplementary Figure 9. XRD pattern (a and b) and UV-vis absorbance spectra (c) of 1000 nm NBG perovskite films with and without Cd^{2+} ions.

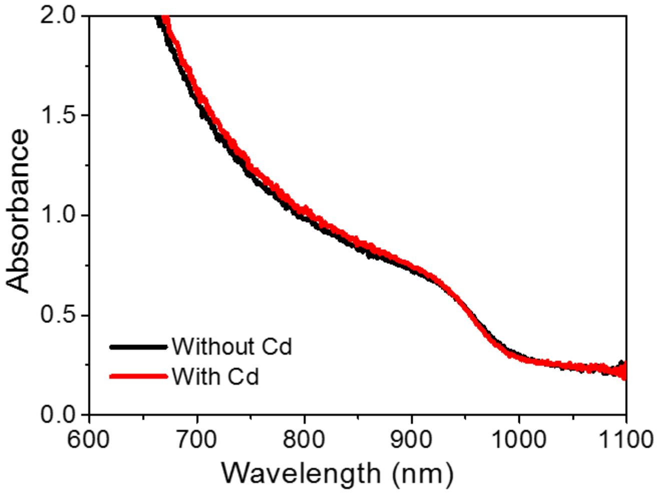

Supplementary Figure 10. UV-vis absorbance spectra of 1000 nm NBG perovskite films with and without Cd^{2+} ions.

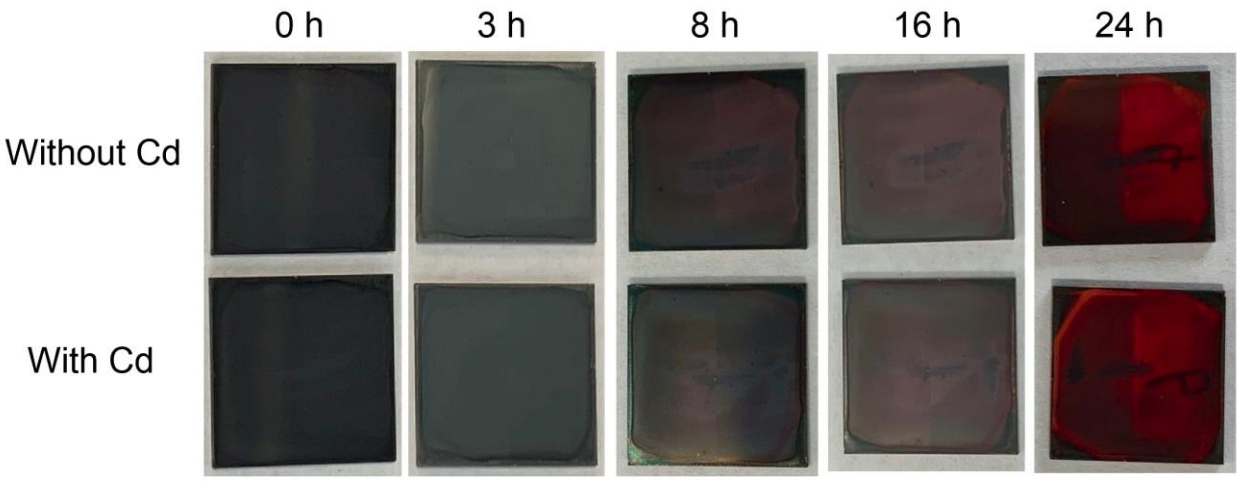

Supplementary Figure 11. Photostability of bare perovskite films with and without Cd^{2+} ions under AM1.5 illumination in ambient condition.

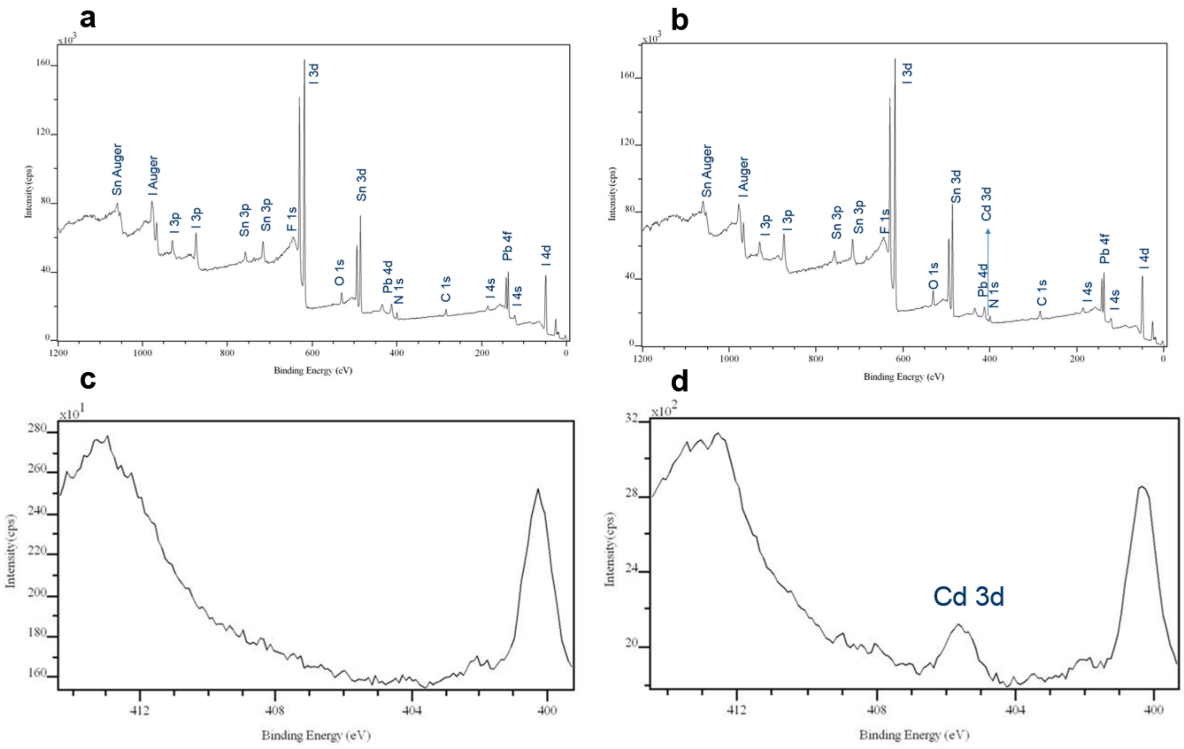

Supplementary Figure 12. XPS spectra of 1000 nm NBG perovskite films with (b, d) and without (a, c) Cd^{2+} ions.

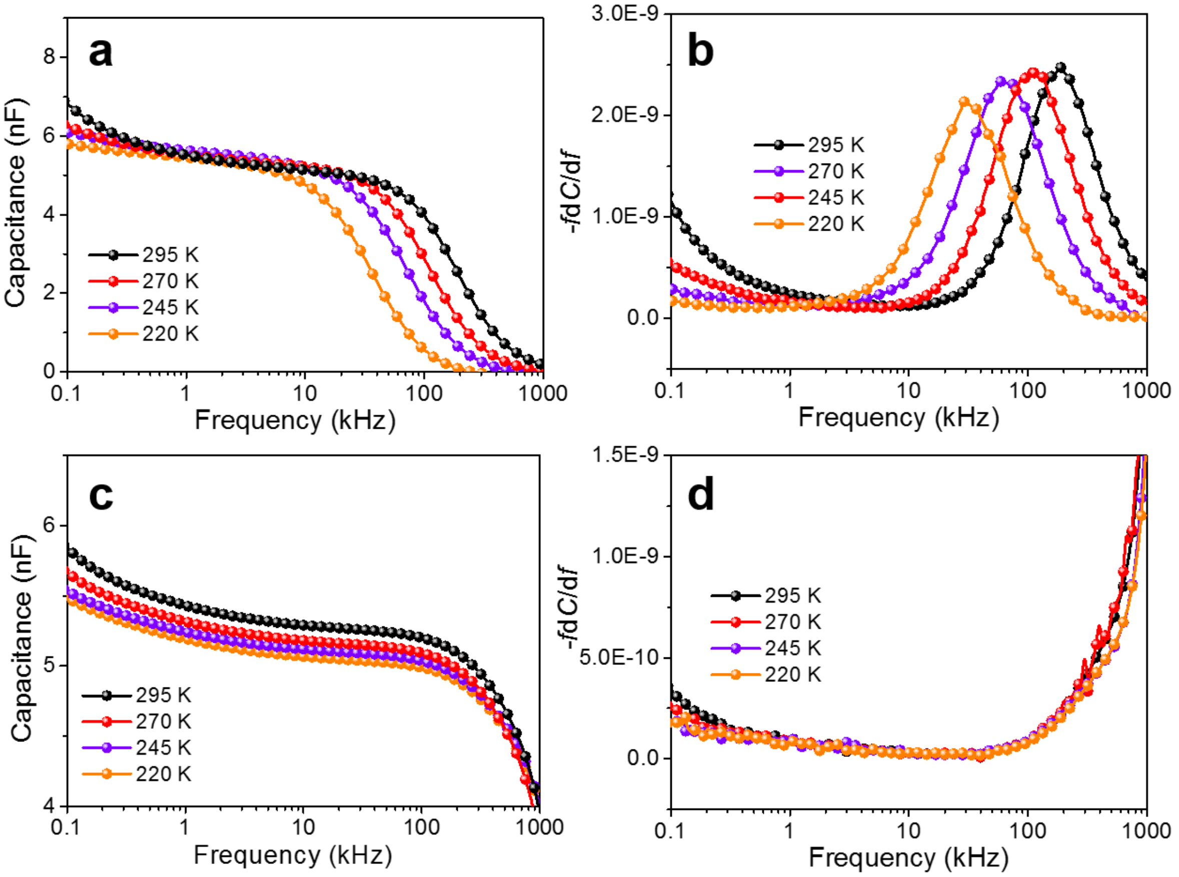

Supplementary Figure 13, **a and b**, Temperature dependent *C*-*f* curves and corresponded -*f*d*C*/d*f* vs *f* curves for Cd-free devices from 220 to 295 K. **c and d,** Temperature dependent *C*-*f* curves and corresponded -*f*d*C*/d*f* vs *f* curves for Cd-containing devices from 220 to 295 K.

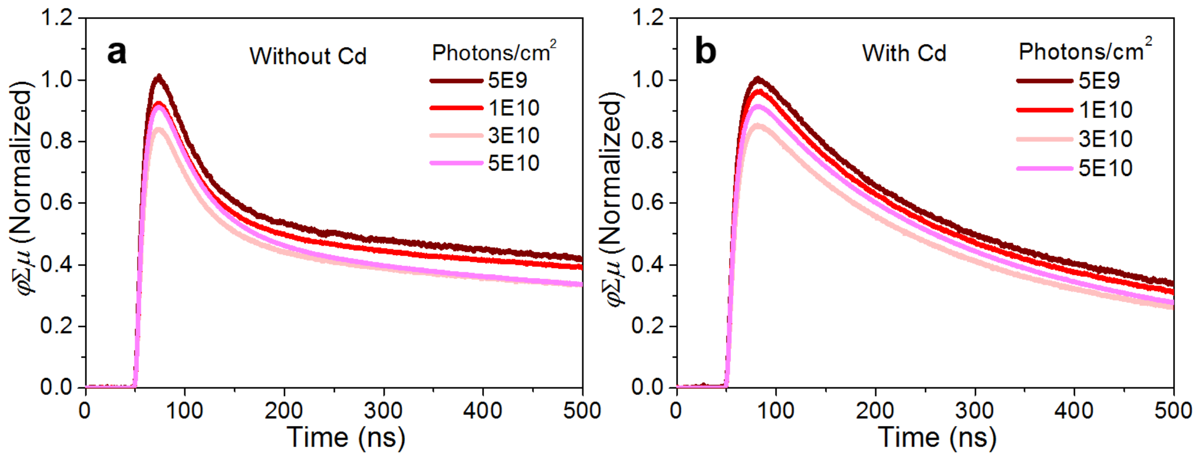

Supplementary Figure 14. TRMC measurement of hole carrier property with a structure of quartz/PCBM /NBG Perovskite films without (a) and with (b) Cd^{2+} ions. The intensities are normalized to the highest peak intensity.

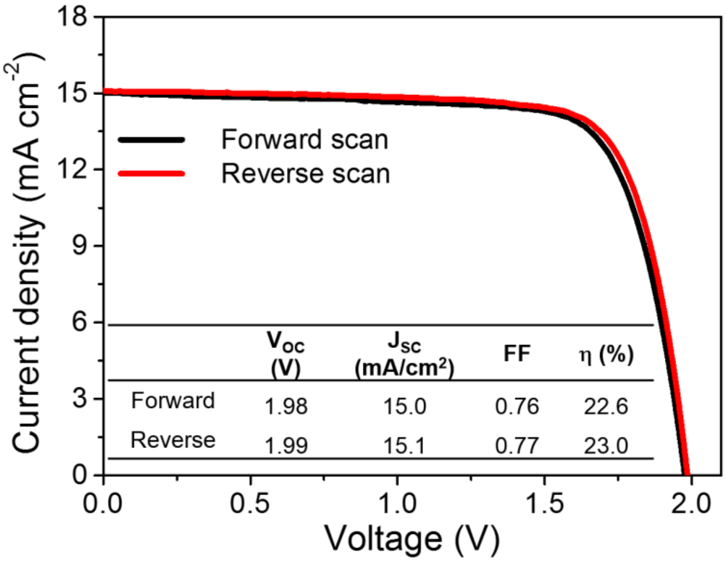

Supplementary Figure 15. Hysteresis study of the perovskite-perovskite tandem solar cells.

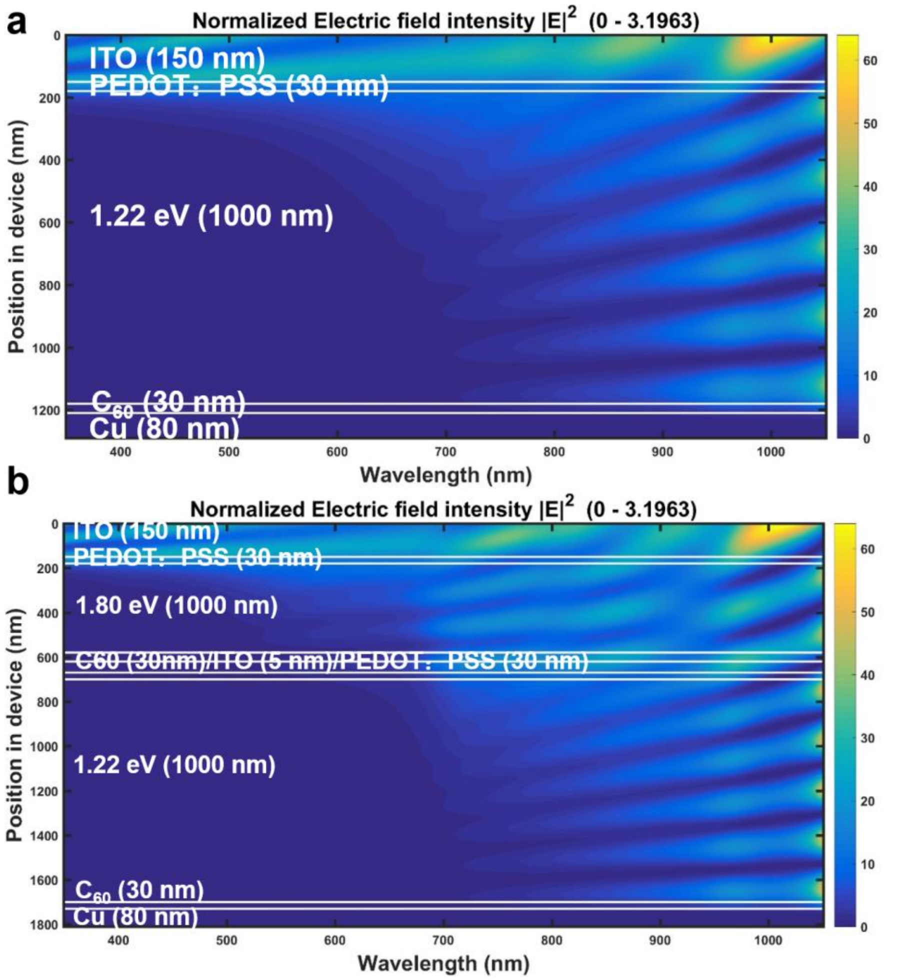

Supplementary Figure 16. Light field distribution of single junction 1.22 eV NBG PSCs and all perovskite tandem solar cells.

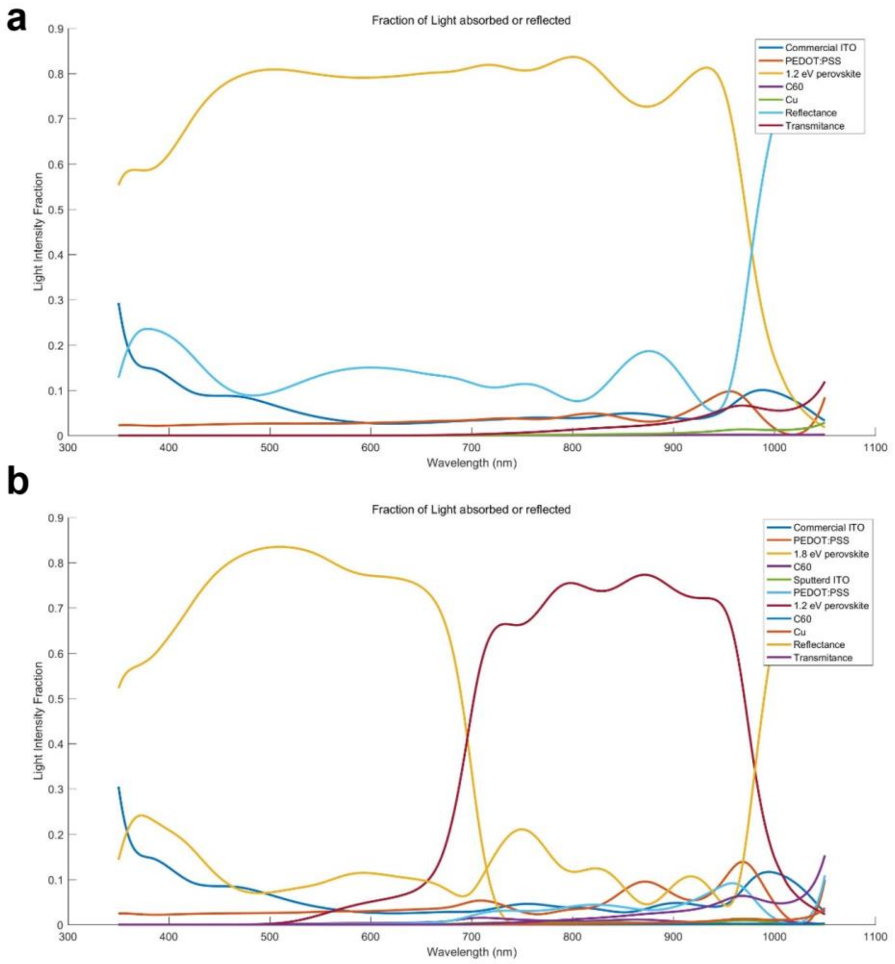

Supplementary Figure 17. Light absorption and reflection of different layers in the tandem solar cells.

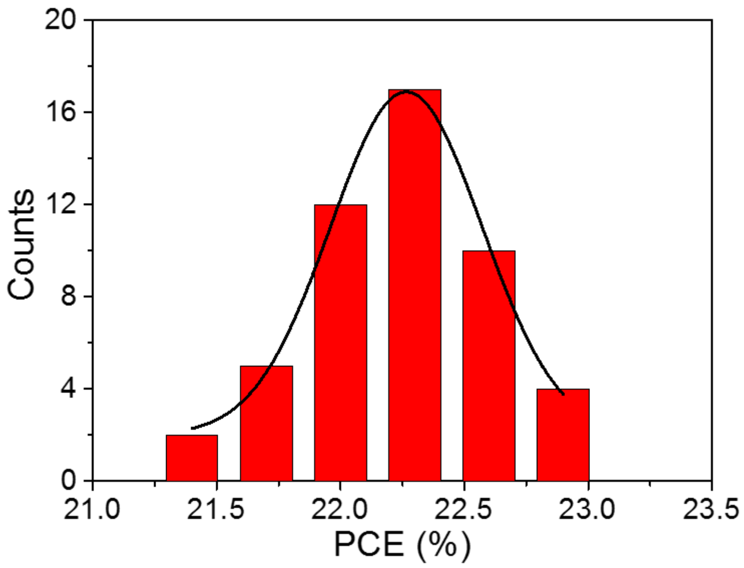

Supplementary Figure 18. Efficiency distribution of 50 perovskite-perovskite tandem solar cells.

Supplementary Table 1. *J-V* characteristics of Cd-free NBG PSCs with different perovskite film thicknesses measured under AM1.5 illumination.

**Thickness (nm)**	**V_{OC} (V)**	**J_{SC} (mA cm^{-2})**	**FF**	**PCE (%)**
370	0.83±0.02 (0.85)	24.7±0.6 (25.4)	0.77±0.02 (0.78)	16.1±0.6 (16.8)
580	0.83±0.02 (0.85)	26.6±0.8 (27.3)	0.76±0.02 (0.78)	17.2±0.6 (18.1)
820	0.82±0.03(0.85)	27.0± 0.6 (27.6)	0.71±0.03 (0.73)	16.5±0.5 (17.1)
1000	0.79±0.03 (0.81)	25.1±1.0 (25.9)	0.64±0.04 (0.67)	13.3±0.6 (14.1)
1140	0.72±0.03 (0.75)	23.4±1.2 (24.5)	0.60±0.03 (0.62)	10.2±0.7 (11.3)

Average values with standard deviation. The data for each sample was obtained from 20 devices. Parameters of the best cell are reported in brackets.

Supplementary Table 2. *J-V* characteristics of NBG PSCs with different molar ratios of Cd^{2+} ions in perovskite film measured under AM1.5 illumination. The thicknesses of perovskite films in all NBG PSCs are 1000 nm.

Content of Cd^{2+} ions	V_{OC} (V)	J_{SC} (mA cm^{-2})	FF	PCE (%)
0%	0.79±0.02 (0.81)	25.1±0.7 (25.9)	0.65±0.03 (0.67)	13.0±0.8 (14.1)
0.01%	0.79±0.03 (0.82)	27.8±0.8 (28.6)	0.71±0.02 (0.71)	14.7±1.2 (16.6)
0.03%	0.83±0.03 (0.85)	29.7±0.6 (30.2)	0.78±0.02 (0.79)	19.4±0.6 (20.3)
0.05%	0.82±0.02 (0.84)	28.7±0.4 (29.2)	0.75±0.03 (0.76)	17.7±0.6 (18.6)
0.07%	0.83±0.02 (0.85)	28.0±0.5 (28.6)	0.73±0.02 (0.73)	17.1±0.5 (17.8)

Average values with standard deviation. The data for each sample was obtained from 20 devices. Parameters of the best cell are reported in brackets.

Supplementary Table 3. *J-V* characteristics of NBG PSCs with 0.03% Cd^{2+} ions in different perovskite film thicknesses measured under AM1.5 illumination.

Thickness (nm)	V_{OC} (V)	J_{SC} (mA cm^{-2})	Integrated J_{SC} from EQE (mA cm^{-2})	FF	PCE (%)
370	0.84±0.02 (0.86)	24.8±0.4 (25.3)	25.5	0.78±0.02 (0.79)	16.4±0.6 (17.2)
580	0.84±0.02 (0.85)	27.0±0.6 (27.4)	27.2	0.77±0.02 (0.79)	17.6±0.6 (18.4)
820	0.84±0.02 (0.85)	28.7±0.9 (29.2)	28.5	0.77±0.02 (0.78)	18.4±0.7 (19.3)
1000	0.83±0.03 (0.85)	29.7±0.6 (30.2)	29.4	0.78±0.02 (0.79)	19.4±0.6 (20.3)
1140	0.8±0.03 (0.82)	27.1±0.6 (27.5)	26.7	0.72±0.03 (0.72)	15.3±0.8 (16.3)

Average values with standard deviation. The data for each sample was obtained from 20 devices. Parameters of the best cell are reported in brackets.

Supplementary Table 4. Performance comparison of reported highly efficient NBG (less than1.3 eV) PSCs with vary perovskite layer thickness.

Composition	Thickness (nm)	J_{SC} (mA cm^{-2})	V_{OC} (V)	FF	PCE (%)	References
$FA_{0.75}Cs_{0.25}Pb_{0.5}Sn_{0.5}I_3$	500	26.7	0. 74	0.71	14.1	1
$(FASnI_3)_{0.6}(MAPbI_3)_{0.4}$	500	26.9	0.80	0.71	15.1	2
$MAPb_{0.5}Sn_{0.5}I_3$	300	26.3	0.75	0.69	13.6	3
$(FASnI_3)_{0.6}(MAPbI_3)_{0.4}$	620	28.7	0.85	0.71	17.5	4
$MAPb_{0.5}Sn_{0.5}I_3$	350	25.5	0.84	0.67	14.4	5
$(FASnI_3)_{0.6}(MAPbI_3)_{0.4}$	750	29.0	0.84	0.74	18.1	6
$MA_{0.4}FA_{0.6}Sn_{0.6}Pb_{0.4}(I_{0.94}Br_{0.06})_3$	620	28.7	0.88	0.75	19.1	7
$(FASnI_3)_{0.6}(MAPbI_3)_{0.4}$	310	27.6	0.84	0.73	17.8	8
$MAPb_{0.5}Sn_{0.5}I_3$	500	26.2	0.87	0.69	15.2	9
$FA_{0.5}MA_{0.45}Cs_{0.05}Pb_{0.5}Sn_{0.5}I_3$ (This work)	1000	30.2	0.85	0.79	20.3	

Supplementary Table 5. Capacitance and *RC* constant of solar cells with different device area in measuring the electron carrier mobility by TPC method.

	Capacitance (nF)	*RC* Constance (ns)
Cd-free devices	2.00	101
	0.98	49
	0.17	8.5
	0.10	5.1
	0.035	1.8
Cd-containing devices	1.50	75
	0.60	30
	0.19	9.5
	0.09	4.5
	0.022	1.1

Supplementary Table 6. Capacitance and *RC* constant of solar cells with different device area in measuring the hole carrier mobility by TPC method.

	Capacitance (nF)	RC Constance (ns)
Cd-free device	3.74	187
	1.34	67
	0.38	19
	0.076	3.8
Cd-containing devices	2.98	149
	0.68	34
	0.3	15
	0.062	3.1

Supplementary Table 7. *J-V* characteristics of 50 perovskite tandem solar cells.

**Samples**	V_{OC} **(V)**	J_{SC} **(mA cm^{-2})**	**FF**	**PCE (%)**
1	1.94	14.9	0.74	21.3
2	1.92	15.2	0.74	21.5
3	1.92	15.1	0.75	21.7
4	1.95	15.0	0.74	21.6
5	1.95	15.3	0.73	21.7
6	1.93	14.2	0.80	21.8
7	1.97	14.9	0.74	21.6
8	1.95	14.5	0.77	21.9
9	1.99	14.7	0.76	22.1
10	1.95	14.8	0.76	22.0
11	1.93	15.2	0.75	22.1
12	1.93	14.3	0.79	21.9
13	1.96	15.2	0.74	22.0
14	1.93	15.5	0.74	22.1
15	1.98	14.9	0.75	22.0
16	1.92	15.0	0.76	21.9
17	1.94	14.2	0.80	22.1
18	1.93	14.5	0.78	21.9
19	1.96	15.3	0.73	22.0
20	1.94	14.9	0.76	21.9
21	1.98	15.2	0.74	22.4
22	1.96	14.9	0.76	22.3
23	1.95	15.0	0.76	22.2
24	2.01	15.4	0.72	22.4

25	1.93	15.5	0.75	22.3
26	1.97	14.9	0.76	22.2
27	1.95	14.8	0.77	22.3
28	1.97	15.3	0.74	22.3
29	1.95	14.8	0.77	22.2
30	1.96	15.2	0.75	22.4
31	1.95	14.9	0.76	22.2
32	1.96	15.4	0.74	22.3
33	1.93	15.1	0.77	22.3
34	1.97	14.9	0.76	22.2
35	1.95	15.4	0.75	22.4
36	1.94	15.3	0.75	22.3
37	1.97	14.9	0.76	22.2
38	1.97	14.9	0.77	22.6
39	1.94	14.7	0.80	22.7
40	1.95	14.8	0.78	22.5
41	1.95	15.2	0.76	22.6
42	2.00	15.5	0.73	22.5
43	1.96	15.2	0.76	22.6
44	1.94	14.9	0.78	22.6
45	1.95	15.0	0.78	22.7
46	1.97	14.7	0.78	22.5
47	1.96	14.8	0.78	22.6
48	1.96	15.2	0.76	22.7
49	1.97	14.9	0.78	22.8
50	1.99	15.1	0.77	23.0

Supplementary references

1 Eperon, G. E. *et al.* Formamidinium lead trihalide: a broadly tunable perovskite for efficient planar heterojunction solar cells. *Energy Environ. Sci.* **7**, 982-988 (2014).

2 Liao, W. *et al.* Fabrication of Efficient Low-Bandgap Perovskite Solar Cells by Combining Formamidinium Tin Iodide with Methylammonium Lead Iodide. *J. Am. Chem. Soc.* **138**, 12360-12363 (2016).

3 Li, Y. *et al.* 50% Sn-Based Planar Perovskite Solar Cell with Power Conversion Efficiency up to 13.6%. *Adv. Energy Mater.* **6**, 1601353 (2016).

4 Zhao, D. *et al.* Low-bandgap mixed tin–lead iodide perovskite absorbers with long carrier lifetimes for all-perovskite tandem solar cells. *Nat. Energy* **2**, 17018 (2017).

5 Rajagopal, A. *et al.* Highly Efficient Perovskite–Perovskite Tandem Solar Cells Reaching 80% of the Theoretical Limit in Photovoltage. *Adv. Mater.* **29**, 1702140 (2017).

6 Zhao, D. *et al.* Efficient two-terminal all-perovskite tandem solar cells enabled by high-quality low-bandgap absorber layers. *Nat. Energy* **3**, 1093-1100 (2018).

7 Li, C. *et al.* Reducing Saturation-Current Density to Realize High-Efficiency Low-Bandgap Mixed Tin–Lead Halide Perovskite Solar Cells. *Adv. Energy Mater.* **9**, 1803135 (2019).

8 Xu, G. *et al.* Integrating Ultrathin Bulk-Heterojunction Organic Semiconductor Intermediary for High-Performance Low-Bandgap Perovskite Solar Cells with Low Energy Loss. *Adv. Funct. Mater.* **28**, 1804427 (2018).

9 Rajagopal, A., Liang, P.-W., Chueh, C.-C., Yang, Z. & Jen, A. K. Y. Defect Passivation via a Graded Fullerene Heterojunction in Low-Bandgap Pb-Sn Binary Perovskite Photovoltaics. *ACS Energy Lett.* **2**, 2531-2539 (2017).

UNC COLLEGE OF ARTS & SCIENCES

THE UNIVERSITY of NORTH CAROLINA at CHAPEL HILL

DEPARTMENT OF CHEMISTRY

CAUDILL AND KENAN LABORATORIES
CAMPUS BOX 3290
CHAPEL HILL, NC 27599-3290

T 919.843.7100
F 919.962.2388

August 16, 2019

To whom it may concern,

Two narrow bandgap perovskite solar cells and two perovksite-perovskite tandem solar cells manufactured by Jinsong Huang group have been tested by Wei You's group of University of North Carolina Chapel Hill with measurement conditions and results as showing below:

Scan conditions: Rate: 0.1 V/s; Dwell time: 0.1 s
Environmental conditions: Temperature: 25 °C; Relative humidity: 0 %
Solar simulator: Type: Oriel 91160, 300 W ; Light intensity: 100 mW/cm^2
Calibration:, Newport calibrating system P/N 5110V.
Number of cells: 4
Solar cell dimension: 2 x 5 mm^2 for all devices
Aperture area: 5.8 mm^2 for all devices

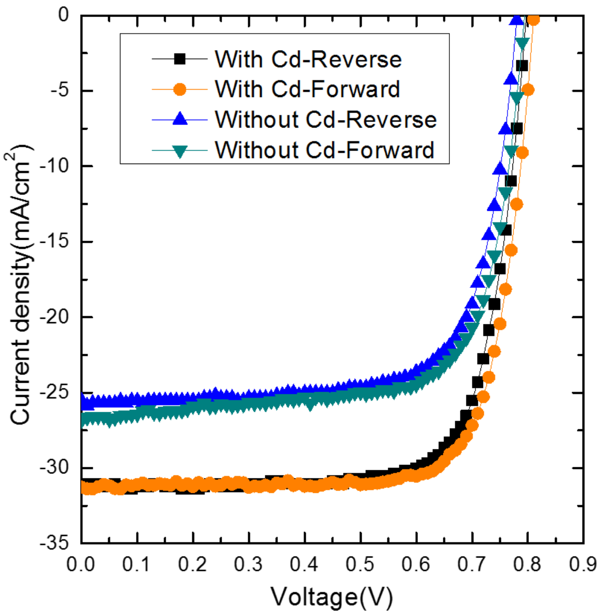

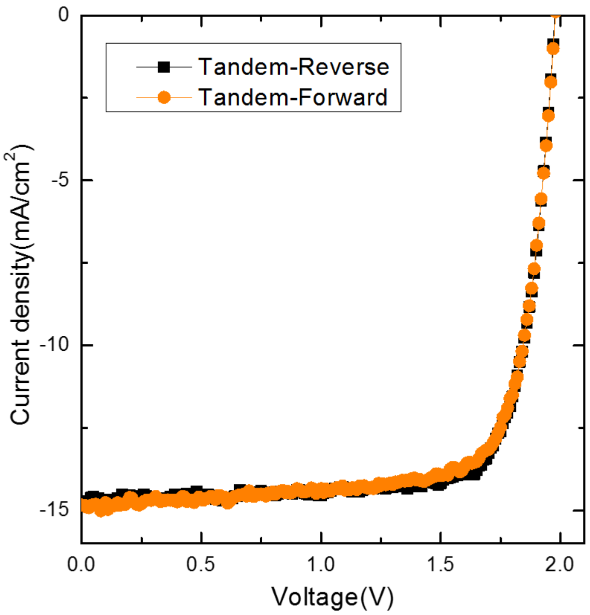

	V_{OC} (V)	J_{SC} (mA/cm^2)	FF	η (%)
With Cd Reverse	0.80	31.21	0.75	18.7
With Cd Forward	0.81	31.26	0.76	19.3
Without Cd Reverse	0.78	25.52	0.73	14.5
Without Cd Forward	0.79	26.84	0.71	15.1

	V_{OC} (V)	J_{SC} (mA/cm^2)	FF	η (%)
Reverse	1.98	14.81	0.78	22.8
Forward	1.98	14.85	0.76	22.4

Tested by:
Jun Hu and Liang Yan

Principle Investigator:

Prof. Wei You
Department of Chemistry and Applied Physical Sciences
University of North Carolina Chapel Hill
Kenan Laboratories C540, Chapel Hill, NC, 27599

Sincerely yours,

Wei You
Professor, FRSC
Department of Chemistry
University of North Carolina at Chapel Hill
Associate Editor, *Polymer Chemistry*, a RSC Journal
Tel: (919) 962-6197 Email: wyou@unc.edu
