# Supplementary material for: Enhancing electron diffusion length in narrow-bandgap perovskites for efficient monolithic perovskite tandem solar cells
Source: Nat Commun. 2019 Oct 3;10:4498. doi: 10.1038/s41467-019-12513-x (PMC6776504; doi:10.1038/s41467-019-12513-x)
Supplement: Supplementary file 2 — Solar Cells Reporting Summary [file 41467_2019_12513_MOESM2_ESM.pdf]

nature research

Corresponding author(s): Jinsong Huang

Solar Cells Reporting Summary

Nature Research wishes to improve the reproducibility of the work that we publish. This form is intended for publication with all accepted papers reporting the characterization of photovoltaic devices and provides structure for consistency and transparency in reporting. Some list items might not apply to an individual manuscript, but all fields must be completed for clarity.

For further information on Nature Research policies, including our data availability policy, see Authors & Referees.

Experimental design

Please check: are the following details reported in the manuscript?

1. Dimensions

Item	Yes	No	Details
Area of the tested solar cells	☒	☐	8.00 mm2
Method used to determine the device area	☒	☐	Defined by the overlap between the pre-patterned ITO and back metal electrode.

2. Current-voltage characterization

Item	Yes	No	Details
Current density-voltage (J-V) plots in both forward and backward direction	☒	☐	Figure S2
Voltage scan conditions<br>*For instance: scan direction, speed, dwell times*	☒	☐	Reverse scan, 0.05 V/s, Dwell time: 0.1 s
Test environment<br>*For instance: characterization temperature, in air or in glove box*	☒	☐	All device are measured in air at room temperature.
Protocol for preconditioning of the device before its characterization	☐	☒	No preconditioning is required before characterization.
Stability of the J-V characteristic<br>*Verified with time evolution of the maximum power point or with the photocurrent at maximum power point; see ref. 7 for details.*	☒	☐	Steady-state output were measured by tracking the maximum power point (Figure 5d and 5f and S3).

3. Hysteresis or any other unusual behaviour

Item	Yes	No	Details
Description of the unusual behaviour observed during the characterization	☒	☐	The efficiency increases in the first several hours during the photostability testing.
Related experimental data	☒	☐	Figure 5f.

4. Efficiency

Item	Yes	No	Details
External quantum efficiency (EQE) or incident photons to current efficiency (IPCE)	☒	☐	Figure 2i and 5e.
A comparison between the integrated response under the standard reference spectrum and the response measure under the simulator	☒	☐	Figure 2i and 5c and 5e.
For tandem solar cells, the bias illumination and bias voltage used for each subcell	☒	☐	The EQE of wide and narrow bandgap sub-cells in tandem cells were measured by respectively exposing the tandem cell under a 470 nm and 940 nm LED lamp for saturating the other junction during measurement.

5. Calibration

Item	Yes	No	Details
Light source and reference cell or sensor used for the characterization	☒	☐	Simulated AM 1.5 G irradiation was produced by a Xenon lamp solar simulator (Oriel 67005, 450 W AAA Solar Simulator). The light intensity was calibrated by a reference solar cell and meter (P/N 5110V, Newport).

Confirmation that the reference cell was calibrated and certified	☒ Yes ☐ No	reference solar cell (P/N 5110V) was certified by Newport.
Calculation of spectral mismatch between the reference cell and the devices under test	☐ Yes ☒ No	A best AAA Solar Simulator is used for JV measurement and the short-circuit current density from JV curves and EQEs are compared.

6. Mask/aperture

Size of the mask/aperture used during testing	☒ Yes ☐ No	6.84 mm2
Variation of the measured short-circuit current density with the mask/aperture area	☐ Yes ☒ No	The efficiency distribution is provided in Figure S18.

7. Performance certification

Identity of the independent certification laboratory that confirmed the photovoltaic performance	☒ Yes ☐ No	The efficiency were tested in third party lab with a test report.
A copy of any certificate(s)<br>*Provide in Supplementary Information*	☒ Yes ☐ No	A test report in another lab is provided

8. Statistics

Number of solar cells tested	☒ Yes ☐ No	50 tandem cells were fabricated and measured to know the efficiency distribution.
Statistical analysis of the device performance	☒ Yes ☐ No	Figure S18.

9. Long-term stability analysis

Type of analysis, bias conditions and environmental conditions<br>*For instance: illumination type, temperature, atmosphere humidity, encapsulation method, preconditioning temperature*	☒ Yes ☐ No	For the long-term photostability measurement in Figure 5f, the tandem devices were encapsulated with Gorilla clear epoxy and slide glass, and connected to a proper resistor so that it can operate at its maximum power point under illumination. Then it was characterized under constant AM 1.5G illumination in ambient condition at room temperature that maintained by a cooling stage.
